# Supplementary material for: Reference curve sampling variability in one–sample log–rank tests
Source: PLoS One. 2022 Jul 21;17(7):e0271094. doi: 10.1371/journal.pone.0271094 (PMC9302761; doi:10.1371/journal.pone.0271094)
Supplement: S1 Appendix — Mathematical statements and corresponding proofs. (PDF) [file pone.0271094.s003.pdf]

One-sample log-rank tests with consideration of reference curve sampling variability: S1 Appendix

Moritz Fabian Danzer[1◑*], Jannik Feld[1◑], Andreas Faldum[1], Rene Schmidt[1],

1 Institute of Biostatistics and Clinical Research, University of Münster, 48149 Münster, Germany

◑These authors contributed equally to this work.
* moritzfabian.danzer@ukmuenster.de

Appendix A: Proof of Distributional Properties

As mentioned in the main text, stochastic processes and martingales are regarded w.r.t. the filtration $(\mathcal{F}_s)_{s\geq 0}$ which is generated by the collected event time data, i.e. for each $s \geq 0$, let $\mathcal{F}_s$ be the σ–algebra generated by

$$\begin{aligned} &I\{T_{x,i} \leq s \wedge C_{x,i}\},\ T_i \cdot I\{T_{x,i} \leq s \wedge C_{x,i}\},\\ &I\{C_{x,i} \leq s \wedge T_{x,i}\},\ C_i \cdot I\{C_{x,i} \leq s \wedge T_{x,i}\}, \end{aligned}$$

for $i \in \mathcal{N}$ and $x = A, B$.

Theorem 1. *Let $s_{max} > 0$ be given s.t. $S_{X_A}(s_{max}) = S_{T_A}(s_{max})S_{C_A}(s_{max}) =: p_0 > 0$ and assume that the null hypothesis $H_0 : \Lambda_A(s) = \Lambda_B(s)$ for all $0 \leq s \leq s_{max}$ is true. Set*

$$\begin{aligned} \widehat{M}_0(s) &:= n_B^{-1/2}\left[N_B(s) - \sum_{i\in\mathcal{N}_B} \widehat{\Lambda}_A(s \wedge X_{B,i})\right] \\ \widehat{\Sigma}_1^2(s) &:= n_B^{-1}N_B(s) + n_B^{-1}n_A^{-1} \sum_{i,j\in\mathcal{N}_B} \widehat{\sigma}_A^2(s \wedge X_{B,i} \wedge X_{B,j}) \end{aligned} \tag{A.1}$$

Then the following is true:

- *$\widehat{M}_0|_{[0,s_{max}]}$ has asymptotically independent increments, i.e. for all $0 \leq s_1 \leq s_2 \leq s_{max}$ and sufficiently large sample size n, the random variables $\widehat{M}_0(s_1)$ and $\widehat{M}_0(s_2) - \widehat{M}_0(s_1)$ are approximately independent.*

- *Pointwise, for each $0 \leq s \leq s_{max}$, we have $\widehat{M}_0(s) \xrightarrow{\mathcal{D}} \mathcal{N}(0, \Sigma^2(s))$ as $n \to \infty$, where $\Sigma(s) := \text{plim}_{n\to\infty}\widehat{\Sigma}(s) = \lim_{n\to\infty} E[\widehat{\Sigma}_1(s)]$ is the large sample limit of $\widehat{\Sigma}_1(s)$ (existing acc. to Lemma 1 below).*

Proof. It is well known that $M_{x,i}(s) := N_{x,i} - \int_0^s I(X_{x,i} \geq u)\lambda_x(u)du$ is a mean–zero $\mathcal{F}_s$–martingale with optional covariation $[M_{x,i}](s) := N_{x,i}$. By independence of the summands, it follows that $M_x(s) := N_x - \int_0^s Y_x(u)\lambda_x(u)du$ is a mean–zero $\mathcal{F}_s$–martingale with optional covariation $[M_x](s) := N_x(s)$. In particular, for any left–continuous $\mathcal{F}_s$–adapted process $H(s)$, $M_H(s) := \int_0^s H(u)dM_A(u)$ is a mean zero $\mathcal{F}_s$–martingale with optional covariation $\int_0^s H^2(u)dN_A(u)$. Choosing $H(u) := J_A(u)/Y_A(u)$ we recover the well–known result that

$M_{J_A/Y_A}(s) \coloneqq \int_0^s \frac{J_A(u)}{Y_A(u)} dM_A(u)$ is a mean zero $\mathcal{F}_s$–martingale with optional covariation $[M_{J_A/Y_A}](s) = \int_0^s \frac{J_A(u)}{Y_A^2(u)} dN_A(u)$. Notice that $M_{J_A/Y_A}(s) = \widehat{\Lambda}_A(s) - \Lambda_A^*(s)$, where $\widehat{\Lambda}_A(s) \coloneqq \int_0^s \frac{J_A(u)}{Y_A(u)} dN_A(u)$ is the Nelsen–Aalen estimate of $\Lambda_A(s)$, and $\Lambda_A^*(s) \coloneqq \int_0^s J_A(u)\lambda_A(u)du$. By independence of the treatment groups it follows that

$$M_i(s) \coloneqq M_{B,i}(s) - M_{J_A/Y_A}(s) \tag{A.2}$$

is a mean–zero $\mathcal{F}_s$–martingale with optional covariation $[M_i](s) \coloneqq [M_{B,i}](s) + [M_{J_A/Y_A}](s)$. Since $\tau_i \coloneqq X_{B,i}$ is an $\mathcal{F}_s$–stopping–time (see Lemma 2 below), we conclude from appeal to the *optional stopping theorem* and *compatibility of stopping with covariation* that the stopped process $M_i^{\tau_i}(s) \coloneqq M_i(s \wedge \tau_i)$ is a mean–zero $\mathcal{F}_s$–martingale with

$$\begin{aligned} &\bullet \quad [M_i^{\tau_i}](s) = [M_i](s \wedge \tau_i) = [M_{B,i}](s \wedge \tau_i) + [M_{J_A/Y_A}](s \wedge \tau_i), \\ &\bullet \quad [M_i^{\tau_i}, M_j^{\tau_j}](s) = [M_{J_A/Y_A}](s \wedge \tau_i \wedge \tau_j) \quad \text{for } i \neq j. \end{aligned} \tag{A.3}$$

To see the last assertion in (A.3), use bilinearity of the covariation operator $[\cdot,\cdot]$ together with $[M_{B,i}^{\tau_i} M_{B,j}^{\tau_j}] = 0$ and $[M_{B,i}^{\tau_i}, M_{J_A/Y_A}^{\tau_j}] = 0$ for $i \neq j$ by independence of the patients and treatment groups, where for any $\mathcal{F}_s$–adapted process Q and any $\mathcal{F}_s$–stopping–time τ we use the common notation $Q^\tau(s) \coloneqq Q(s \wedge \tau)$.

We are finally interested in the large sample properties of the mean zero $\mathcal{F}_s$–martingale

$$\widehat{M}(s) \coloneqq n_B^{-1/2} \sum_{i \in \mathcal{N}_B} M_i^{\tau_i}(s). \tag{A.4}$$

First notice that the jumpsize of $\widehat{M}$ is bounded by $2n_B^{-1/2}$ and thus vanishes in the large sample limit $n \to \infty$, because the jump sizes of $M_{B,i}$ and M_{J_A/Y_A} are bounded by 1, as no two event indicators $N_{x,i}$ and $N_{x',j}$ jump simultaneously a.s.. Making use of $N_{B,i}(s \wedge \tau_i) = N_{B,i}(s)$ (see Lemma 3 below) and noticing that $n_A \cdot [M_{J_A/Y_A}](s) \equiv \widehat{\sigma}_A^2(s)$, some algebra shows that $\widehat{M}$ has optional covariation $[\widehat{M}](s) = \widehat{\Sigma}_1^2(s)$. So, by Lemma 1 below, $[\widehat{M}](s)$ converges pointwise in s in probability to the strictly increasing, deterministic function $\Sigma^2(s) = \lim_{n\to\infty} E[\widehat{\Sigma}_1^2(s)]$. All in all, we conclude from appeal to Rebolledo's martingale central limit theorem that $\widehat{M}$ converges on $[0, s_{\max}]$ in distribution to a mean zero Gaussian martingale $M^{(\infty)}$ with independent increments and variance function $\Sigma^2(s)$.

To finish the proof, it suffices to notice that the processes $\widehat{M}$ from (A.4) and $\widehat{M}_0$ from (A.1) coincide in the limit when null hypothesis $H_0 : \Lambda_A = \Lambda_B$ hold true. □

Corrolary 1. *Consider the assumptions of Theorem 1 and let*

$$\widehat{\Sigma}_2^2(s) \coloneqq n_B^{-1} \sum_{i \in \mathcal{N}_B} \widehat{\Lambda}_A(s \wedge X_{B,i}) + n_B^{-1} n_A^{-1} \sum_{i,j \in \mathcal{N}_B} \widehat{\sigma}_A^2(s \wedge X_{B,i} \wedge X_{B,j}) \tag{A.5}$$

then it holds $\text{plim}_{n\to\infty} \widehat{\Sigma}_2(s) = \Sigma(s)$.

Proof. *Since* $\widehat{M}_0(s) \xrightarrow{\mathcal{D}} \mathcal{N}(0, \Sigma^2(s))$ *it holds* $\text{plim}_{n\to\infty} n^{-1/2} \widehat{M}_0(s) = 0$. *Thus the equality* $\widehat{\Sigma}_2^2(s) = \widehat{\Sigma}_1^2(s) + n^{-1/2}\widehat{M}_0(s)$ *yields the assertion with use of Slutskys theorem.*

Theorem 2. *Fix* $s_{max} > 0$ *as in theorem 1. Under the contiguous alternatives* $\Lambda_B(\cdot) = \omega_n \Lambda_A(\cdot)$ *with* $\omega_n = \exp(-n^{-1/2}\gamma)$ *for some* $\gamma \geq 0$, *the process* $\widehat{M}_0$ *defined in*

(A.1) converges on $[0, s_{max}]$ *in distribution to a Gaussian process with independent increments, drift function* $\mu(s) \coloneqq -\gamma\sqrt{\frac{\pi}{1+\pi}}\int_0^\infty F_{T_A}(s \wedge u) f_{C_A}(u)du$ *and variance function* $\Sigma^2(s)$ *from (A.14).*

Proof. Under the contiguous alternatives, the difference between the mean–zero martingale $\widehat{M}$ from (A.4) and $\widehat{M}_0$ is

$$\begin{aligned}\widehat{M}_0 - \widehat{M} &= n_B^{-1/2} \sum_{i \in \mathcal{N}_B} [\Lambda_B(s \wedge X_{B,i}) - \Lambda_A(s \wedge X_{B,i})] \\ &= n_B^{1/2}[1 - \exp(n^{-1/2}\gamma)] \cdot n_B^{-1} \sum_{i \in \mathcal{N}_B} \Lambda_B(s \wedge X_{B,i}).\end{aligned} \tag{A.6}$$

As $n \to \infty$, the first factor converges to $-\gamma\sqrt{\frac{\pi}{1+\pi}}$. Since $\Lambda_B \approx \Lambda_A$ under the contiguous alternatives when n is large, the second converges in probability to $E[\Lambda_A(s \wedge X_{A,1})]$ by law of large numbers as $n \to \infty$, which in turn coincides with $E[N_{A,1}(s)] = \int_0^\infty F_{T_A}(s \wedge u) f_{C_A}(u)du$ due to the martingale property of $M_{A,1}$. So the assertion follows from Theorem 1 and appeal to Slutsky's theorem. □

Lemma 1. *Let* $s_{max} > 0$ *be given s.t.* $S_{X_A}(s_{max}) = S_{T_A}(s_{max})S_{C_A}(s_{max}) \eqqcolon p_0 > 0$. *Let* $\widehat{\Sigma}_1$ *be the process defined in (A.1). Then there is a strictly increasing, deterministic function* $\Sigma(s)$ *with* $\Sigma(0) = 0$ *such that pointwise for each* $0 \leq s \leq s_{max}$*, we have* $\widehat{\Sigma}_1(s) \xrightarrow{\mathcal{P}} \Sigma(s)$ *as* $n \to \infty$. *More specifically,* $\Sigma(s) = \lim_{n\to\infty} E[\widehat{\Sigma}_1(s)]$.

Proof. For this proof, we introduce the following abbreviations:

$$\begin{aligned}\Theta &\coloneqq n_B^{-1} n_A^{-1} \sum_{i,j \in \mathcal{N}_B} \widehat{\sigma}_A^2(s \wedge X_{B,i} \wedge X_{B,j}), \\ \Psi_{N_A}(s) &\coloneqq \int_0^s \frac{J_A(u)}{Y_A^2(u)} dN_A(u), \\ \Psi_{M_A}(s) &\coloneqq \int_0^s \frac{J_A(u)}{Y_A^2(u)} dM_A(u), \\ \Psi_{\Lambda_A}(s) &\coloneqq \int_0^s \frac{J_A(u)}{Y_A(u)} \lambda_A(u)du.\end{aligned} \tag{A.7}$$

Since $n_B^{-1} N_B(s) \xrightarrow{\mathcal{P}} E[N_{B,1}(s)]$ as $n \to \infty$ by law of large numbers, it remains to prove that $\Theta \xrightarrow{\mathcal{P}} \lim_{n\to\infty} E[\Theta]$ as $n \to \infty$. The proof decomposes into several steps. From appeal to triangle inequality we conclude that for any $\varepsilon > 0$

$$P\left(|\Theta - \lim_{n\to\infty} E[\Theta]| \geq 2\varepsilon\right) \leq P\left(|\lim_{n\to\infty} E[\Theta] - E[\Theta]| \geq \varepsilon\right) + P\left(|\Theta - E[\Theta]| \geq \varepsilon\right). \tag{A.8}$$

By Lemma 6, $\lim_{n\to\infty} E[\Theta]$ exists, i.e. the first summand on the right hand side of equation (A.8) vanishes in the limit $n \to \infty$. By conditioning on the outcomes in group B, the second summand can be rewritten as

$$\begin{aligned}P\left(|\Theta - E[\Theta]| \geq \varepsilon\right) &= E[P\left(|\Theta - E[\Theta]| \geq \varepsilon | X_B\right)] \\ &= \int_{[0,\infty)^{n_B}} P\left(|\Theta - E[\Theta]| \geq \varepsilon | X_B = x_B\right) \cdot f_{X_B}(x_B)dx_B\end{aligned}$$

where $X_B = (X_{B,1}, \ldots, X_{B,n_B})$ and $f_{X_B} = \prod_{i=1}^{n_B} f_{X_{B,1}}(x_{B,i})$ as the observations from group B are independent and identically distributed. Now, if $P\left(|\Theta - E[\Theta]| \geq \varepsilon | \, X_B = x_B\right) \leq c(n_A)$ for some function c which does only depend on n_A and not on x_B with $c(n_A) \to 0$ as $n_A \to \infty$, we also have $P\left(|\Theta - E[\Theta]| \geq \varepsilon\right) \to 0$ as $n \to \infty$. We will prove this uniform convergence next.

By Chebyshev's inequality, the Cauchy-Schwarz inequality and since $\Psi_{M_A}(s) = \Psi_{N_A}(s) - \Psi_{\Lambda_A}(s)$ we have for any $x_B \in [0, \infty)^{n_B}$ and any $\varepsilon > 0$

$$\begin{aligned}
P(|\Theta - E[\Theta]| \geq \varepsilon | X_B = x_B) &\leq \frac{\mathrm{Var}[\Theta | X_B = x_B]}{\varepsilon^2} \\
&= \mathrm{Var}[n_B^{-1} n_A^{-1} \sum_{i,j \in \mathcal{N_B}} \widehat{\sigma}_A^2(s \wedge x_{B,i} \wedge x_{B,j})] \\
&\leq \frac{1}{n_A^2} \cdot \sum_{i,j \in \mathcal{N}_B} \mathrm{Var}\left[\int_0^{s \wedge x_{B,i} \wedge x_{B,j}} n_A \cdot \frac{J_A(u)}{Y_A^2(u)} dN_A(u)\right] \\
&\leq \pi^2 \sup_{s^\star \in [0, s_{\max}]} \mathrm{Var}\left[\int_0^{s^\star} n_A \cdot \frac{J_A(u)}{Y_A^2(u)} dN_A(u)\right] \\
&\leq \pi^2 \Bigg(\sup_{s^\star \in [0, s_{\max}]} \sqrt{\mathrm{Var}[n_A \cdot \Psi_{M_A}(s^\star)]} \\
&\qquad + \sup_{s^\star \in [0, s_{\max}]} \sqrt{\mathrm{Var}[n_A \cdot \Psi_{\Lambda_A}(s^\star)]} \Bigg)^2
\end{aligned} \tag{A.9}$$

where $\pi = n_B / n_A$ is the prefixed treatment group allocation ratio. Notice that the third inequality holds because x_B is a fixed value and not a random variable. The fourth inequality holds as $\mathrm{Var}[A + B] \leq (\sqrt{\mathrm{Var}[A]} + \sqrt{\mathrm{Var}[B]})^2$ by the Cauchy-Schwarz inequality for $\mathrm{Cov}[A, B]$ for two real-valued random variables A and B. To finish the proof, we show that $\sup_{s^\star \in [0, s_{\max}]} \mathrm{Var}[n_A \cdot \Psi_{M_A}(s^\star)] \to 0$ (Step I) and $\sup_{s^\star \in [0, s_{\max}]} \mathrm{Var}[n_A \cdot \Psi_{\Lambda_A}(s^\star)] \to 0$ (Step II) as $n_A \to \infty$.

Proof of Step I:

$$\begin{aligned}
\mathrm{Var}\left[n_a \cdot \Psi_{\Lambda_A}(s)\right] = n_A^2 \cdot \mathrm{Var}\left[\int_0^s \frac{J_A(u)}{Y_A(u)} \lambda_A(u) \, du\right] \\
= n_A^2 \cdot \int_0^s \int_0^s \lambda_A(u) \lambda_A(v) \cdot \underbrace{\mathrm{Cov}\left[\frac{J_A(u)}{Y_A(u)}, \frac{J_A(v)}{Y_A(v)}\right]}_{\leq \sup_{s^\star \in [0, s_{\max}]} \mathrm{Var}\left[\frac{J_A(s^\star)}{Y_A(s^\star)}\right]} \; dudv
\end{aligned}$$

For any $s \in [0, s_{\max}]$, we also have

$$\begin{aligned}
\mathrm{Var}\left[\frac{J_A(s)}{Y_A(s)}\right] &= E\left[\left(\frac{J_A(s)}{Y_A(s)} - E\left[\frac{J_A(s)}{Y_A(s)}\right]\right)^2\right] \\
&\leq E\left[\left(\frac{J_A(s)}{Y_A(s)} - \frac{1}{n_A \cdot S_{X_A}(s)}\right)^2\right] \\
&\leq \frac{1}{n_A^2} \left| E\left[\frac{n_A^2 \cdot J_A(s)}{Y_A^2(s)} - \frac{1}{S_{X_A}(s)^2}\right]\right| + \frac{2}{n_A^2 \cdot S_{X_A}(s)} \left| E\left[\frac{n_A \cdot J_A(s)}{Y_A(s)} - \frac{1}{S_{X_A}(s)}\right]\right|
\end{aligned}$$

The first inequality holds because for any random variable, its expectation minimizes the mean squared error. For both summands we can apply the results from Lemma 4.2 (i) of [2] as the probability that $Y_A(s) = 0$ goes to zero uniformly on $[0, s_{\max}]$ to get

$$\mathrm{Var}\left[\frac{J_A(s)}{Y_A(s)}\right] \leq \frac{K}{n_A^3} + \frac{2K}{n_A^3 \cdot p_0^3}.$$

We can finally plug this estimate in to obtain

$$\sup_{s^\star \in [0, s_{\max}]} \mathrm{Var}\left[n_A \cdot \Psi_{\Lambda_A}(s^\star)\right] \leq \Lambda_A^2(s_{\max}) \cdot \left(\frac{K}{n_A} + \frac{2K}{n_A \cdot p_0^3}\right)$$

which goes to zero on $[0, s_{\max}]$ as $n_A \to \infty$.

Proof of Step II: $\Psi_{M_A}(s)$ is a mean zero $\mathcal{F}_s$–martingale since $M_A(s)$ is so. Consequently, $\Psi_{M_A}^2(s) - [\Psi_{M_A}](s)$ is a mean zero $\mathcal{F}_s$–martingale, where $[\Psi_{M_A}](s) = \int_0^s \frac{J_A(u)}{Y_A^4(u)} dN_A(u)$ is the optional covariation of $\Psi_{M_A}(s)$. Thus

$$\begin{aligned}
\mathrm{Var}\left[\Psi_{M_A}(s)\right] &= E\left[\Psi_{M_A}^2(s)\right] = E\left[[\Psi_{M_A}](s)\right] = E\left[\int_0^s \frac{J_A(u)}{Y_A^4(u)} dN_A(u)\right] \\
&= E\left[\int_0^s \frac{J_A(u)}{Y_A^3(u)} \lambda_A(u) du\right] = \int_0^s E\left[\frac{J_A(u)}{Y_A^3(u)}\right] \lambda_A(u) du \\
&\leq \int_0^s \frac{4^3}{n_A^3 \cdot S_{X_A}^3(u)} \lambda_A(u) du \leq \frac{4^3}{n_A^3 \cdot p_0^3} \cdot \Lambda_A(s_{\max}).
\end{aligned} \tag{A.10}$$

where the first inequality from the third row follows from Lemma 1 from [2]. Those inequalities hold for all $s \leq s_{\max}$. We thus conclude that

$$\sup_{s^\star \in [0, s_{\max}]} \mathrm{Var}\left[n_A \cdot \Psi_{M_A}(s^\star)\right] \leq n_A^{-1} \Lambda_A(s_{\max}) \cdot \frac{4^3}{p_0^3} \xrightarrow[n_A \to \infty]{} 0 \tag{A.11}$$

for any $s \leq s_{\max}$ which finishes the proof. $\square$

Lemma 2. *$\tau_i \coloneqq X_{B,i}$ is an $\mathcal{F}_s$–stopping–time.*

Proof. $\{\tau_i > s\} = \{T_{B,i} \wedge C_{B,i} > s\} = \{T_{B,i} > s\} \cap \{C_{B,i} > s\} \in \mathcal{F}_s$ as s can be replaced by $s \wedge C_{B,i}$ resp. $s \wedge T_{B,i}$ in the last equation. $\square$

Lemma 3. *For any two random variables S and T we have $I(T \leq S) = I(T \leq S \wedge T)$. In particular, $N_{B,i}(s \wedge \tau_i) = N_{B,i}(s)$ for the $\mathcal{F}_s$–stopping–time $\tau_i \coloneqq X_{B,i}$.*

Proof. $\{T \leq T \wedge S\} = \{T \leq T \wedge S, T \leq S\} \cup \{T \leq T \wedge S, T > S\} = \{T \leq T, T \leq S\} \cup \{T \leq S, T > S\} = \{T \leq S\}$. $\square$

Lemma 4. *Let $s_{max} \geq s > 0$ and consider*

$$\widehat{\sigma}_x^2(s) \coloneqq n_x \cdot \int_0^s \frac{J_x(u)}{Y_x^2(u)} dN_x(u) \;\equiv\; n_x \cdot \sum_{\substack{i \in \mathcal{N}_x, \\ N_{x,i}(s) = 1}} \frac{J_x(T_{x,i})}{Y_x^2(T_{x,i})}.$$

Then in probability as $n \to \infty$

$$\widehat{\sigma}_x^2(s) \xrightarrow{\mathcal{P}} \sigma_x^2(s) \coloneqq \int_0^s \frac{\lambda_x(u)}{S_{T_x}(u) \cdot S_{C_x}(u)} du, \tag{A.12}$$

where S_{T_x} (S_{C_x}) is the survival function of the time to event $T_{x,i}$ (time to censoring $C_{x,i}$) in treatment group $x = A, B$.

Proof. This can easily be shown with Hellands proposition [3] and using the fact that $Y_x(u)/n_x \xrightarrow{\mathcal{P}} y_x(u) \equiv S_{T_x}(u) \cdot S_{C_x}(u)$. $\square$

Lemma 5. *Let $X_{x,i} \coloneqq T_{x,i} \wedge C_{x,i}$. Then, for any $i \neq j$, the density $f_{X_{x,i}}(u)$ and survival function $S_{X_{x,i}}(u)$ of $X_{x,i}$ as well as the density $f_{X_{x,i} \wedge X_{x,j}}(u)$ of $X_{x,i} \wedge X_{x,j}$ are*

$$\begin{aligned} f_{X_{x,i}}(u) &= f_{T_x}(u)S_{C_x}(u) + S_{T_x}(u)f_{C_x}(u) \\ S_{X_{x,i}}(u) &= S_{T_x}(u)S_{C_x}(u) \\ f_{X_{x,i} \wedge X_{x,j}}(u) &= 2[f_{T_x}(u)S_{C_x}(u) + S_{T_x}(u)f_{C_x}(u)]S_{T_x}(u)S_{C_x}(u). \end{aligned} \tag{A.13}$$

where f_{T_x} and S_{T_x} (f_{C_x} and S_{C_x}) are density and survival function of the time to event $T_{x,i}$ (time to censoring $C_{x,i}$) in treatment group $x = A, B$.

Proof. Follows from elementary calculation with probability distributions using the independence of $T_{x,i}$, $T_{x,j}$, $C_{x,i}$ and $C_{x,j}$ for $i \neq j$. $\square$

Lemma 6. *Let $\widehat{\Sigma}_1$ the process defined in (A.1). Then, pointwise in s, the limit $\Sigma(s) \coloneqq \lim_{n\to\infty} E[\widehat{\Sigma}_1(s)]$ exists. Under the contiguous alternatives $\Lambda_B(\cdot) = \omega_n \Lambda_A(\cdot)$ with $\omega_n = \exp(-n^{-1/2}\gamma)$ for some $\gamma \geq 0$, $\Sigma(s)$ amounts to*

$$\begin{aligned} \Sigma^2(s) = &\int_0^\infty F_{T_A}(s \wedge u) f_{C_A}(u) du \\ &+ 2\pi \cdot \int_0^\infty \sigma_A^2(s \wedge u)[f_{T_A}(u)S_{C_A}(u) + S_{T_A}(u)f_{C_A}(u)]S_{T_A}(u)S_{C_A}(u)du \end{aligned} \tag{A.14}$$

where f_{T_x}, F_{T_x}, S_{T_x} (f_{C_x}, F_{C_x}, S_{C_x}) are density, distribution function and survival function of the time to event $T_{x,i}$ (time to censoring $C_{x,i}$) in treatment group $x = A, B$, where $\sigma_A^2(\cdot)$ is the function from (A.12), and $\pi = n_B/n_A$ the prefixed treatment arm allocation ratio.

Proof. Since each of the families of random variables $\{N_{B,i}(s)\}_{i \in \mathcal{N}_B}$, $\{\widehat{\sigma}_x^2(s \wedge X_{x,i})\}_{i \in \mathcal{N}_B}$ and $\{\widehat{\sigma}_x^2(s \wedge X_{x,i} \wedge X_{x,j})\}_{i \neq j \in \mathcal{N}_B}$ is identically distributed, we have

$$E[\widehat{\Sigma}_1^2(s)] = E[N_{B,1}(s)] + \frac{n_B}{n_B n_A} E[\widehat{\sigma}_A^2(s \wedge X_{B,1})] + \frac{n_B(n_B - 1)}{n_B n_A} E[\widehat{\sigma}_A^2(s \wedge X_{B,1} \wedge X_{B,2})]. \tag{A.15}$$

Thus, the limit $\Sigma(s) = \lim_{n\to\infty} E[\widehat{\Sigma}_1(s)]$ exists. Now additionally assume the contiguous alternatives. Then $\lim_{n\to\infty} E[N_{B,1}(s)] = E[N_{A,1}(s)]$, because $\lim_{n\to\infty} \Lambda_B = \Lambda_A$ under the contiguous alternatives. Moreover, by independence of the treatment groups and by (A.12), we conclude that

$$\begin{aligned} \lim_{n\to\infty} E[\widehat{\sigma}_A^2(s \wedge X_{B,i} \wedge X_{B,j})] &= \lim_{n\to\infty} E_u[E[\widehat{\sigma}_A^2(s \wedge X_{B,i} \wedge X_{B,j}) | X_{B,i} \wedge X_{B,j} = u]] \\ &= \lim_{n\to\infty} \int_0^\infty E[\widehat{\sigma}_A^2(s \wedge u)] f_{X_{B,i} \wedge X_{B,j}}(u) du \\ &= \int_0^\infty \sigma_A^2(s \wedge u) \lim_{n\to\infty} f_{X_{B,i} \wedge X_{B,j}}(u) du \\ &= \int_0^\infty \sigma_A^2(s \wedge u) f_{X_{A,i} \wedge X_{A,j}}(u) du. \end{aligned} \tag{A.16}$$

where the third equality holds by dominated convergence under application of the estimate from Lemma 1 in [1] and the convergence of the expectation holds by Lemma 4.2 in [2]. In particular, the second summand on the right hand side of (A.15) vanishes as $n \to \infty$. So we are done by supplying the explicit value of the density function $f_{X_{A,i} \wedge X_{A,j}}$ from (A.13). Notice that the last equality in (A.16) holds, because $\lim_{n\to\infty} \Lambda_B = \Lambda_A$ under the contiguous alternatives. □

Appendix B:

As indicated in the main text, the type I error rate inflation is driven by the ratio of the standard deviation of the standard one-sample log-rank test $\Sigma_{\mathrm{OSLR}}(s_{\max})$ and the asymptotic standard deviation of the corrected method $\Sigma(s_{\max})$. If these values are known, one can compute the ratio

$$R \coloneqq \Sigma_{\mathrm{OSLR}}(s_{\max})/\Sigma(s_{\max}). \tag{B.1}$$

In practice, both values need to be estimated. The corresponding ratio is then given by

$$\widehat{R}_i \coloneqq \widehat{\Sigma}_{\mathrm{OSLR},i}(s_{\max})/\widehat{\Sigma}_i(s_{\max}).$$

with $i \in \{1, 2\}$. Using our previous results, we can see that (B.1) amounts to

$$R = \int_0^\infty \frac{\Sigma_{\mathrm{OSLR}}(s_{\max})}{\sqrt{\Sigma^2_{\mathrm{OSLR}}(s_{\max}) + 2\pi \cdot \int_0^\infty \sigma_A^2(s_{\max} \wedge u) f_{X_{B,i}\wedge X_{B,j}} du}}$$

If the accrual and censoring mechanism in groups A and B is the same, we have under the null hypothesis that

$$\begin{aligned}
&\Sigma^2_{\mathrm{OSLR}}(s_{\max}) \\
=&\int_0^\infty F_{T_B}(s_{\max} \wedge u) f_{C_B}(u) du \\
=&\int_0^{s_{\max}} f_{T_B}(u) S_{C_B}(u) du \\
=&\int_0^{s_{\max}} \frac{f_{T_B}(u)}{S^2_{T_B}(u) S_{C_B}(u)} S^2_{T_B}(u) S^2_{C_B}(u) du \\
=&\int_0^{s_{\max}} \sigma_B^2(u) 2 S_{T_B}(u) f_{T_B}(u) S^2_{C_B}(u) du + \int_0^{s_{\max}} \sigma_B^2(u) 2 S_{C_B}(u) f_{C_B}(u) S^2_{T_B}(u) du \\
&+ \left[\sigma_B^2(u) S^2_{T_B}(u) S^2_{C_B}(u)\right]_0^{s_{\max}} \\
=&\int_0^\infty \sigma_B^2(s_{\max} \wedge u) 2 S_{T_B}(u) f_{T_B}(u) S^2_{C_B}(u) du \\
&+ \int_0^\infty \sigma_B^2(s_{\max} \wedge u) 2 S_{C_B}(u) f_{C_B}(u) S^2_{T_B}(u) du \\
=&2 \int_0^\infty \sigma_A^2(s_{\max} \wedge u) f_{X_{B,i}\wedge X_{B,j}} du
\end{aligned}$$

by application of partial integration and some rearrangements of the terms. Consequently, we obtain

$$R = \sqrt{\frac{1}{1+\pi}}$$

for that special case.

For the a priori assessment of the type I error rate inflation, we required some approximations. In particular, we approximated

- $E\left[2\cdot\Phi\left(\sqrt{\widehat{R}_i}\cdot z_{\frac{\alpha}{2}}\right)\right]$ by $2\cdot\Phi\left(E\left[\sqrt{\widehat{R}_i}\right]\cdot z_{\frac{\alpha}{2}}\right)$,
- $E\left[\sqrt{\widehat{R}_i}\right]$ by $\sqrt{E\left[\widehat{R}_i\right]}$ and
- $E[\widehat{\Sigma}_{\mathrm{OSLR},i}(s_{\max})/\widehat{\Sigma}_i(s_{\max})]$ by $E[\widehat{\Sigma}_{\mathrm{OSLR},i}(s_{\max})]/E[\widehat{\Sigma}_i(s_{\max})]$.

All of these approximations are motivated by first order Taylor expansions. For a function $f\colon \mathbb{R}^d \to \mathbb{R}$, the first order expansion around a point $a \in \mathbb{R}^d$ is given by

$$f(x) = f(a) + \nabla f(a)(x-a) + R$$

where R is a remainder term containing higher-order terms. By using this expansion for the functions $f(x) = 2\cdot\Phi(x\cdot z_{\frac{\alpha}{2}})$, $f(x)=\sqrt{x}$ resp. $f(x) = x_1/x_2$ around the points $E[\sqrt{\widehat{R}_i}]$, $E[\widehat{R}_i]$ resp. $(E[\widehat{\Sigma}_{\mathrm{OSLR},i}(s_{\max})], E[\widehat{\Sigma}_i(s_{\max})])$, neglecting the remainder and taking expectations, we obtain the above-mentioned approximations. Notice that, these approximations can be improved using higher order terms of the Taylor expansion. In particular, this may also mitigate the bias caused by these approximations for small sample sizes. In particular, this is the case for the second approximation as $E[\sqrt{\widehat{R}_i}] \leq \sqrt{E[\widehat{R}_i]}$ by Jensen's inequality.

In what follows we derive the asymptotic expectation of $\widehat{\Sigma}_i(s_{\max})$ as shown in the Case Study. Firstly, $E[\widehat{\Sigma}_i^2(s_{\max})]$ can be decomposed into

$$n_B^{-1}E[N_B(s_{\max})] + n_B^{-1}n_A^{-1}\sum_{i,j\in\mathcal{N}_B} E[\widehat{\sigma}_A^2(s_{\max}\wedge X_{B,i}\wedge X_{B,j})]$$

resp.

$$n_B^{-1}E\left[\sum_{i\in\mathcal{N}_B}\widehat{\Lambda}_A(s_{\max}\wedge X_{B,i})\right] + n_B^{-1}n_A^{-1}\sum_{i,j\in\mathcal{N}_B} E[\widehat{\sigma}_A^2(s_{\max}\wedge X_{B,i}\wedge X_{B,j})]$$

where the first summand is given by $\int_0^\infty F_{T_B}(s_{\max}\wedge u)dF_{C_B}(u)$ for the first equation resp. converges to this expression for the second sum. For the second summand, we have

$$\begin{aligned}
&n_B^{-1}n_A^{-1}\sum_{i,j\in\mathcal{N}_B} E[\widehat{\sigma}_A^2(s_{\max}\wedge X_{B,i}\wedge X_{B,j})]\\
=&n_A^{-1}E[\widehat{\sigma}_A^2(s_{\max}\wedge X_{B,1})] + (n_B-1)n_A^{-1}E[\widehat{\sigma}_A^2(s_{\max}\wedge X_{B,1}\wedge X_{B,2})]\\
\to&\pi\cdot E[\widehat{\sigma}_A^2(s_{\max}\wedge X_{B,1}\wedge X_{B,2})]
\end{aligned}$$

as $n\to\infty$. The expectation can be computed by plugging in the density from (A.13). Then, one obtains by

$$\begin{aligned}
&\int_0^\infty \widehat{\sigma}_A^2(s_{\max}\wedge u)dF_{X_{B,1}\wedge X_{B,2}}(u)\\
=&2\int_0^\infty \widehat{\sigma}_A^2(s_{\max}\wedge u)S_{T_B}^2(u)S_{C_B}(u)dF_{C_B}(u) + 2\int_0^\infty \widehat{\sigma}_A^2(s_{\max}\wedge u)S_{T_B}(u)S_{C_B}^2(u)dF_{T_B}(u).
\end{aligned}$$

Appendix C: Additional Simulation Results

Table 1. Empirical type I error rates under consideration of sampling variability for $\kappa = 0.5$

	$\pi = 1$		$\pi = 1/2$		$\pi = 1/4$		$\pi = 1/8$		$\pi = 1/16$	
n_B	$\widehat{\alpha}$	$\widehat{R}_i$	$\widehat{\alpha}$	$\widehat{R}_i$	$\widehat{\alpha}$	$\widehat{R}_i$	$\widehat{\alpha}$	$\widehat{R}_i$	$\widehat{\alpha}$	$\widehat{R}_i$
using $\widehat{\Sigma}_{\text{OSLR},1}$ as variance estimator										
25	0.156	0.697	0.109	0.810	0.083	0.889	0.069	0.939	0.062	0.967
50	0.161	0.702	0.109	0.813	0.081	0.891	0.068	0.941	0.059	0.968
100	0.165	0.705	0.109	0.815	0.080	0.893	0.064	0.942	0.058	0.969
200	0.164	0.706	0.108	0.816	0.079	0.894	0.065	0.942	0.057	0.970
using $\widehat{\Sigma}_{\text{OSLR},2}$ as variance estimator										
25	0.175	0.697	0.117	0.810	0.085	0.889	0.069	0.939	0.061	0.967
50	0.170	0.702	0.113	0.813	0.083	0.891	0.068	0.941	0.059	0.968
100	0.169	0.705	0.112	0.815	0.081	0.893	0.066	0.942	0.059	0.969
200	0.166	0.706	0.110	0.816	0.080	0.894	0.064	0.942	0.057	0.970

(i) Empirical type I error rates $\widehat{\alpha}$ of test procedure (8) when used for testing $H_0 : \Lambda_B = \Lambda_A$, and (ii) median factor $\widehat{R}_i$ as in (11) by which the true standard deviation of the one–sample log–rank statistic $\widehat{M_0}$ is underestimated when ignoring the reference curve sampling variability for different parameter constellations of practical relevance. Survival times were Weibull distributed with shape parameter $\kappa = 0.5$ and 1–year survival rate $S_1 = 0.5$ in the historic control group A and the new treatment group B. Theoretical two–sided significance level: 5%. Underlying sample size of group B is n_B with allocation ratio $\pi = n_B/n_A$ between new and historic groups.

Table 2. Empirical one-sided type I error rates under consideration of sampling variability for $\kappa = 0.5$

	$\pi = 1$		$\pi = 1/2$		$\pi = 1/4$		$\pi = 1/8$		$\pi = 1/16$	
n_B	$\widehat{\alpha}_{\text{inf}}$	$\widehat{\alpha}_{\text{sup}}$	$\widehat{\alpha}_{\text{inf}}$	$\widehat{\alpha}_{\text{sup}}$	$\widehat{\alpha}_{\text{inf}}$	$\widehat{\alpha}_{\text{sup}}$	$\widehat{\alpha}_{\text{inf}}$	$\widehat{\alpha}_{\text{sup}}$	$\widehat{\alpha}_{\text{inf}}$	$\widehat{\alpha}_{\text{sup}}$
using $\widehat{\Sigma}_{\text{OSLR},1}$ as variance estimator										
25	0.099	0.057	0.077	0.031	0.062	0.021	0.053	0.016	0.048	0.014
50	0.096	0.065	0.071	0.039	0.055	0.027	0.047	0.021	0.042	0.017
100	0.093	0.071	0.065	0.044	0.049	0.030	0.041	0.023	0.038	0.021
200	0.089	0.075	0.061	0.047	0.046	0.033	0.038	0.026	0.034	0.023
using $\widehat{\Sigma}_{\text{OSLR},2}$ as variance estimator										
25	0.060	0.115	0.041	0.076	0.029	0.057	0.022	0.047	0.019	0.042
50	0.067	0.104	0.044	0.069	0.031	0.052	0.025	0.043	0.021	0.037
100	0.071	0.097	0.047	0.065	0.033	0.048	0.027	0.039	0.024	0.035
200	0.074	0.093	0.048	0.062	0.034	0.046	0.027	0.037	0.024	0.033

(i) Empirical one-sided type I error rates $\widehat{\alpha}_{\text{inf}}$ and $\widehat{\alpha}_{\text{sup}}$ of test procedures (9) when used for testing $H_{0,\text{inf}}$ and $H_{0,\text{sup}}$ for different parameter constellations of practical relevance. Survival times were Weibull distributed with shape parameter $\kappa = 0.5$ and 1–year survival rate $S_1 = 0.5$ in the historic control group A and the new treatment group B. Theoretical one–sided significance level: 2.5%.Underlying sample size of group B is n_B with allocation ratio $\pi = n_B/n_A$ between new and historic groups.

Table 3. Apriori estimated type I error rates under consideration of sampling variability for $\kappa = 0.5$

	$\pi = 1$		$\pi = 1/2$		$\pi = 1/4$		$\pi = 1/8$		$\pi = 1/16$	
n_B	$\widehat{\alpha}_{\text{pre}}$	R_{pre}	$\widehat{\alpha}_{\text{pre}}$	R_{pre}	$\widehat{\alpha}_{\text{pre}}$	R_{pre}	$\widehat{\alpha}_{\text{pre}}$	R_{pre}	$\widehat{\alpha}_{\text{pre}}$	R_{pre}
25	0.162	0.714	0.109	0.819	0.079	0.895	0.065	0.943	0.057	0.970
50	0.164	0.710	0.109	0.818	0.079	0.895	0.065	0.943	0.057	0.970
100	0.165	0.709	0.109	0.817	0.080	0.895	0.065	0.943	0.057	0.970
200	0.165	0.708	0.109	0.817	0.080	0.895	0.065	0.943	0.057	0.970

(i) Median a priori estimates of type I error rate $\widehat{\alpha}_{\text{pre}}$ (see equation (15)) of test procedure (8) when used for testing $H_0 : \Lambda_B = \Lambda_A$, and (ii) median a priori estimates of underestimation of the standard deviation R_{pre} (see equation (14)) of the one–sample log–rank statistic $\widehat{M_0}$ when ignoring the reference curve sampling variability for different parameter constellations of practical relevance. Survival times were Weibull distributed with shape parameter κ and 1–year survival rate $S_1 = 0.5$ in the historic control group A. Underlying sample size of $n_A = n_B/\pi$ with allocation ratio π.

Table 4. Empirical type I error rates under consideration of sampling variability for $\kappa = 2$

	$\pi = 1$		$\pi = 1/2$		$\pi = 1/4$		$\pi = 1/8$		$\pi = 1/16$	
n_B	$\widehat{\alpha}$	$\widehat{R}_i$	$\widehat{\alpha}$	$\widehat{R}_i$	$\widehat{\alpha}$	$\widehat{R}_i$	$\widehat{\alpha}$	$\widehat{R}_i$	$\widehat{\alpha}$	$\widehat{R}_i$
using $\widehat{\Sigma}_{\text{OSLR},1}$ as variance estimator										
25	0.129	0.686	0.089	0.799	0.068	0.880	0.058	0.931	0.052	0.960
50	0.144	0.694	0.098	0.806	0.074	0.886	0.061	0.936	0.056	0.964
100	0.152	0.699	0.103	0.810	0.075	0.889	0.062	0.939	0.055	0.967
200	0.158	0.702	0.105	0.813	0.077	0.892	0.063	0.941	0.056	0.969
using $\widehat{\Sigma}_{\text{OSLR},2}$ as variance estimator										
25	0.162	0.686	0.111	0.799	0.081	0.880	0.069	0.931	0.062	0.960
50	0.162	0.694	0.108	0.806	0.080	0.886	0.067	0.936	0.060	0.964
100	0.161	0.699	0.108	0.810	0.080	0.889	0.065	0.939	0.058	0.967
200	0.162	0.702	0.108	0.813	0.078	0.892	0.065	0.941	0.057	0.969

(i) Empirical type I error rates $\widehat{\alpha}$ of test procedure (8) when used for testing $H_0 : \Lambda_B = \Lambda_A$, and (ii) median factor $\widehat{R}_i$ as in (11) by which the true standard deviation of the one–sample log–rank statistic $\widehat{M_0}$ is underestimated when ignoring the reference curve sampling variability for different parameter constellations of practical relevance. Survival times were Weibull distributed with shape parameter $\kappa = 2$ and 1–year survival rate $S_1 = 0.5$ in the historic control group A and the new treatment group B. Theoretical two–sided significance level: 5%. Underlying sample size of group B is n_B with allocation ratio $\pi = n_B/n_A$ between new and historic groups.

Table 5. Empirical one-sided type I error rates under consideration of sampling variability for $\kappa = 2$

n_B	$\pi = 1$		$\pi = 1/2$		$\pi = 1/4$		$\pi = 1/8$		$\pi = 1/16$	
	$\widehat{\alpha}_{\text{inf}}$	$\widehat{\alpha}_{\text{sup}}$	$\widehat{\alpha}_{\text{inf}}$	$\widehat{\alpha}_{\text{sup}}$	$\widehat{\alpha}_{\text{inf}}$	$\widehat{\alpha}_{\text{sup}}$	$\widehat{\alpha}_{\text{inf}}$	$\widehat{\alpha}_{\text{sup}}$	$\widehat{\alpha}_{\text{inf}}$	$\widehat{\alpha}_{\text{sup}}$
using $\widehat{\Sigma}_{\text{OSLR},1}$ as variance estimator										
25	0.064	0.064	0.053	0.035	0.044	0.023	0.040	0.018	0.037	0.015
50	0.072	0.072	0.055	0.043	0.045	0.029	0.039	0.022	0.036	0.020
100	0.075	0.077	0.056	0.047	0.043	0.032	0.036	0.025	0.033	0.022
200	0.078	0.080	0.056	0.050	0.042	0.034	0.035	0.027	0.032	0.024
using $\widehat{\Sigma}_{\text{OSLR},2}$ as variance estimator										
25	0.039	0.122	0.030	0.081	0.022	0.059	0.020	0.049	0.018	0.044
50	0.051	0.111	0.036	0.073	0.028	0.053	0.023	0.044	0.021	0.039
100	0.059	0.102	0.042	0.066	0.031	0.049	0.025	0.040	0.022	0.035
200	0.066	0.097	0.045	0.063	0.033	0.045	0.027	0.038	0.024	0.033

(i) Empirical one-sided type I error rates $\widehat{\alpha}_{\text{inf}}$ and $\widehat{\alpha}_{\text{sup}}$ of test procedures (9) when used for testing $H_{0,\text{inf}}$ and $H_{0,\text{sup}}$ for different parameter constellations of practical relevance. Survival times were Weibull distributed with shape parameter $\kappa = 0.5$ and 1–year survival rate $S_1 = 2$ in the historic control group A and the new treatment group B. Theoretical one–sided significance level: 2.5%. Underlying sample size of group B is n_B with allocation ratio $\pi = n_B/n_A$ between new and historic groups.

Table 6. Apriori estimated type I error rates under consideration of sampling variability for $\kappa = 2$

n_B	$\pi = 1$		$\pi = 1/2$		$\pi = 1/4$		$\pi = 1/8$		$\pi = 1/16$	
	α_{pre}	R_{pre}	α_{pre}	R_{pre}	α_{pre}	R_{pre}	α_{pre}	R_{pre}	α_{pre}	R_{pre}
25	0.149	0.736	0.104	0.829	0.078	0.899	0.064	0.944	0.057	0.971
50	0.156	0.724	0.106	0.824	0.079	0.897	0.064	0.944	0.057	0.970
100	0.160	0.716	0.108	0.821	0.079	0.896	0.064	0.943	0.057	0.970
200	0.163	0.712	0.109	0.819	0.079	0.895	0.065	0.943	0.057	0.970

(i) Median a priori estimates of type I error rate α_{pre} (see equation (8)) of test procedure (15) when used for testing $H_0 : \Lambda_B = \Lambda_A$, and (ii) median a priori estimates of underestimation of the standard deviation R_{pre} (see equation (14)) of the one–sample log–rank statistic $\widehat{M_0}$ when ignoring the reference curve sampling variability for different parameter constellations of practical relevance. Survival times were Weibull distributed with shape parameter κ and 1–year survival rate $S_1 = 0.5$ in the historic control group A. Underlying sample size of $n_A = n_B/\pi$ with allocation ratio π.

References

1. McKeague IW , Utikal KJ. Inference for a nonlinear counting process regression model. Ann Stat. 1990; 18(3):1172–1187.

2. Aalen OO. Nonparametric inference in connection with multiple decrement models. Scand J Stat. 1976; 3(1):15–27.

3. Andersen PK, Borgan O, Gill RD, Keiding N. Statistical Models Based on Counting Processes. New York: Springer Series in Statistics; 1993.
