# Supplementary material for: Reference curve sampling variability in one–sample log–rank tests
Source: PLoS One. 2022 Jul 21;17(7):e0271094. doi: 10.1371/journal.pone.0271094 (PMC9302761; doi:10.1371/journal.pone.0271094)
Supplement: Attachment — Submitted filename: Answers to comments.pdf [file pone.0271094.s004.pdf]

Answer to editor and reviewer comments

concerning the manuscript 'One-sample log-rank tests with consideration of reference curve sampling variability' (PONE-D-21-25863)

January 27, 2022

In the review of the article 'One-sample log-rank tests with consideration of reference curve sampling variability' by J Feld, MF Danzer, A Faldum and R Schmidt, submitted to PLOS ONE (PONE-D-21-25863), the editor suggested splitting the manuscript into two new manuscripts. The first of these papers should describe the problem of uncertainty of the reference survival curve which is usually estimated from a historic control cohort and should address and study the resulting amount of type I error rate inflation of the one-sample log-rank test. As encouraged by the editor, we prepared and herewith submit such a manuscript. Following the editor's request, we took all comments of the three reviewers concerning this first part of our work into account. Detailed answers to these comments follow below. We also give references to the corresponding passages in the new manuscript which have been changed accordingly.

1 Comments of the editor

Comment: *[...] present the conclusion that a two-sample approach is required to compare the survival of the patients of a single-arm study to a reference survival curve (e.g. by means of the usual log-rank test).*
Answer: As requested, we added the conclusion that the two-sample log-rank test is to be used when the historic control group is too small.
Manuscript passage: l. 197-199 (discussion of simulation results), l. 314-320 (Discussion)

Comment: *You should add to this paper a thorough discussion of the problem of confounding in such non-randomized comparisons.*
Answer: We thank the editor for this suggestion. In the discussion section, we added a paragraph concerning this topic.
Manuscript passage: l. 16-19 (Introduction), l. 342-349 (Discussion)

2 Comments of Reviewer #1

Comment: *The Time-window and s_{max}: In survival analysis, there is often a discussion about the time window of the analysis. E.g. the variance of the Kaplan-Meier-estimator 'explodes' near to the maximum point, sometimes, denoted as τ ($\tau = \sup\{t : P(X > t) > 0\}$ where $X = \min(C, T)$ is the observed time). Depending on the research question it is, thus, only possible to analyse the data from a smaller time window. A prominant example is the restricted mean survival time, the mean over a pre-specified time-window $[0, \tau_0]$.*
My question: When I understand the authors correctly, this is also not the case for their statistic (compare to (6) where the endpoint of the time window is infinite). HOWEVER, the theory in the supplement is only developed for a time window $[0, s_{max}]$, where s_{max} is smaller than τ, so the theory and the proposal does not perfectly fit. In particular, the statment in (6) was not proven in the supplement. My personal feeling is that (6) can be proven similar as it can be done for the log-rank tests, but here some more work by the authors need to be done. This would solve my doubt immediately. Otherwise the statments in (6) and before (6) need to be adjusted to fit what is proven in the supplement.
Answer: In the former version of the manuscript, we have used the formal argument "∞" to show that all data from both groups are included in the test statistics. However, as mentioned by the reviewer, the theory can only be applied if the time window of observation in the experimental group is smaller than τ, i.e. the maximal time window of observation in the control group.
Hence, we added a reference to the theoretical statement in the Appendix and a note clarifying the required condition $\mathbb{P}[X > s_0] > 0$. Nevertheless, we have kept the formal argument "∞" in formulas (5), (7) and (8) to show that no data from any group is discarded in the final analysis if the condition addressed is complied with.
Manuscript passage: l. 111-113

Comment: *In line 74, s_{max} is introduced as some positive, maximum observation time. Here, I would also prefer a more concrete statement, i.e. that $s_{max} < \tau$ or verbalize the conditions in the supplement (they are not that difficult). Otherwise, s_{max} might be interpreted as the maximum observation time from the data set, i.e. as a data-dependent quantity, which is not allowed, right?*
Answer: In the introduction of the one-sample log-rank test and its practical implementation, in which the reference cumulative hazard is replaced by an estimate from a historic control group, we verbalized the condition mentioned by the reviewer. We interpret $[0, s_{\max}]$ as a prefixed observation horizon that is chosen in advance independent from the data.
Manuscript passage: l. 93-96

Comment: *Quote from the Discussion section: 'Admittedly, our simulations suggest that it may be advisable to compare the data of a historical control cohort with the new data in a single-arm Phase II trial via the two-sample log-rank test if one wants to account for sampling variability of the reference curve or in case*

of allocation ratios close to 1.'
When I started to read the paper, I also thought about why not using a two-sample test for this problem (at the end we have to groups, right? the new data and the reference group). The author convinced me that the use of the one-sided test is not adequat. But why not use the two-sample test? A more in-depth discussion of this issue would be very much appreciated.
Answer: The discussion section now contains a paragraph in which we clearly state, that the two-sample test shall be applied in settings where the one-sample test is clearly inappropriate. Appropriateness of the one-sample log-rank test depends in particular on the size of the control as compared to the active treatment group. Additionally, we discuss knwon problems of the two-sample log-rank test in case of small sample sizes and unbalanced groups and the additional problem of confounding.
Manuscript passage: l. 197-199 (discussion of simulation results), l. 314-320, 342-349 (Discussion)

Comment: *π, the treatment group allocation ratio is kept fix as $n \to \infty$, right? I did not find a statement about it, but the argumentation in the proofs suggest so.*
Answer: This is right, thank you. It is now stated upon introduction of π in the manuscript.
Manuscript passage: l. 53-54

Comment: *The supplement need to be carefully revised. The literature references and references to the main paper appear as (??) or [??].*
Answer: We apologize for this, the supplement has been revised and missing references were added.
Manuscript passage: S1 Appendix

Comment: *It is rather unusal to use σ and not σ^2 for the variance or its estimator, see e.g. (4). Moreover, it is not consistent with the other estimator Σ in (5) where the square is used (e.g. $\widehat{\Sigma}^2$). But this is rather a personal view, the authors can also keep it as it is.*
Answer: The reviewer is right. The notation has been revised to be consistent throughout the manuscript.
Manuscript passage: equation (4)

Comment: *In the design for study I line 176, the hazard ratio w is introduced. But in the simulaltions for Study I just $w = 1$ is considered, right? This should be stated in the text.*
Answer: In the new manuscript, we focus on type I error rate inflation of the one-sample log-rank test as suggestes by the editor. The respective section has therefore been removed from the present paper and will be content of an upcoming paper.

Comment: *First row in (A.3), the minus sign should be replaced by +, right? (compare to the second line on the same page).*
Answer: This is correct. The typo has been corrected.
Manuscript passage: equation (A.3) in S1 Appendix

Comment: *Lemma 4. For which s is the result valid?*
Answer: Thank you for this remark. It is valid for $s_0 \geq s$ which is now also stated in Lemma 4.
Manuscript passage: Lemma 4 in S1 Appendix

Comment: *Not so important: but the last calculation in the supplement is not needed. The result follows immediately from the respective density in (A.12).*
Answer: The calculation has been shortened by referring to the density given in (A.12).
Manuscript passage: Appendix B in S1 Appendix

Comment: *2.Equation in (2), $N_{x,i}$ instead of N_i.*
Answer: The typo has been corrected.
Manuscript passage: equation (2) in S1 Appendix

3 Comments of Reviewer #2

Comment: *The assumption that there are no two counting processes that jump simultaneously should be stated explicitly somewhere on p. 2 or 3. Is this assumption satisfied for the data set analyzed further back in the paper? If not, how was this problem solved? A suggestion: could a Greenwood-type variance estimator for the studentization be the solution? See for instance the (unpublished) note by Jon Wellner, https://sites.stat.washington.edu/jaw/COURSES/580s/582/HO/Greenwood.pdf*
Answer: We thank the reviewer for this important comment. From our point of view, the underlying distribution of the event time under consideration is non-atomic. Therefore, there should be no counting processes that jump simultaneously. However, tied event times may occur in clinical practice for logistical reasons when only coarsened data (e.g. rounded to number of days) is recorded. This is also the case for the data set analyzed in the case study. The "true" event times would not be tied with finer temporal resolution. Nevertheless, the variance estimator

$$\check{\sigma}_x^2(s) := n_x \cdot \int_0^s \frac{J_x(u)(Y_x(u) - \Delta N_x(u))}{Y_x^3(u)} dN_x(u). \qquad (*)$$

for the Nelson-Aalen estimator as presented e.g. in formula (4.1.7) of Andersen's text book "Statistical Models Based on Counting Processes" might be used instead of the estimator given in (4) if tied events shall be taken into account. For the following reasons, we did not explicitly account for ties in our manuscript:

1. The "classical" variance estimator (see formula (4) of our manuscript) is an upper bound for the variance estimator $(*)$ as $(Y_x(u) - \Delta N_x(u))/Y_x(u) \leq 1$ as long as $Y_x(u) \geq 0$. Thus, the approximations of type I error rates based on the variance estimator from (4) ignoring ties also serves as an upper bound of the type I error rate inflation in the presence of ties.

2. The computations presented here (in particular those of the case study) shall serve as a "rule of thumb" to estimate type I error rate inflation during the planning phase of the one-sample test. Even if the survival dates can be rounded, the number of tied event times is likely to be quite small if the historic data comes from a reasonably designed study, which is why the two estimators are unlikely to differ much. For the sake of simplicity of our work we thus focus on the setting of no ties.

3. In order to support these arguments, we repeated the computations of our case study under replacement of $\widehat{\sigma}^2$ from (4) by $\check{\sigma}^2$ from $(*)$. The results can be found in the following plot. The original curves are given in blue. The curves for which $\check{\sigma}^2$ was used are given in red. The "uncorrected"

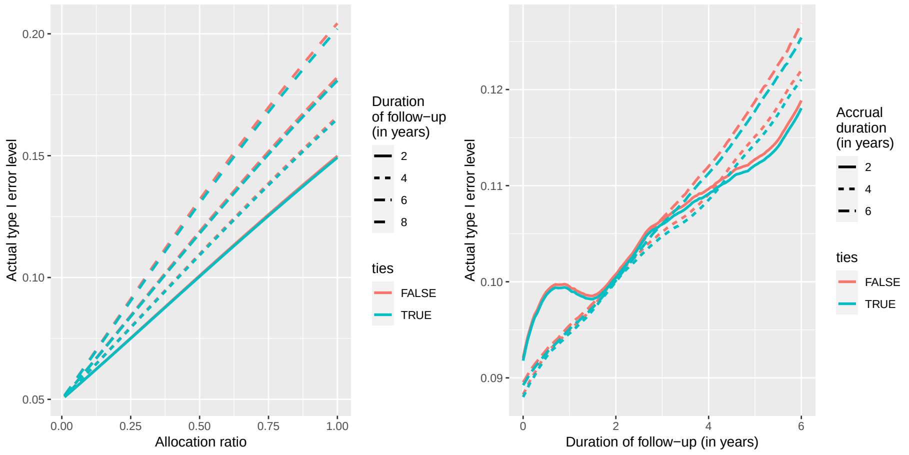

version serves as an upper bound and the difference between two curves belonging to one pair is very small. The relative difference maximum absolute difference is 0.2 percentage points (left plot) and 0.14 percentage points (right plot), respectively. In both plots, the maximum relative difference amounts to 1.2%.

However, we thank the reviewer very much for this comment and in our forthcoming work in which we will present the emerging test statistic as an alternative two-sample survival test, we will take such considerations into account. Because in this case, strict control of error rates is of an even higher importance.

<u>Comment:</u> *Some few typos across the paper; also, on p. 6, l. 175, why is 'acc.' the only abbreviated word in the whole article?*

Answer: We removed all typos we could find and we wrote out the word addressed by the reviewer.

Comment: *p. 3, Section 1.1: the stochastic process M_0 is not nicely motivated. As a sanity-check, it might help the reader if first the classical log-rank test statistic (or its numerator) is introduced such that M_0 could be deduced.*
Answer: In the revised manuscript, we introduce M_0 as the (unstandardized) test statistic of a one-sample log-rank test and present the corresponding testing problem. Hereafter, we present the usual procedure of replacing the (unknown) reference cumulative hazard function by an estimate to point out the problem that is being investigated.
Manuscript passage: Section Motivation, in particular l. 76-99

Comment: *I would like to see the computations that led to the required sample sizes $n = r \cdot a$ in both simulation studies.*
Answer: In the first simulation study, the sample size was fixed to the values $n \in \{100, 500, 1000\}$ for all scenarios. We realize that the sample size of the second simulation study of the old paper was not easily understandable from the given despcriptions. We will explain this in more detail in the upcoming second part of the paper, where this table will be presented.

Comment: *In the supplement S1: missing references, "(??)" and "[?]", should be filled. Because of this, parts of the proofs could not be checked.*
Answer: We apologize for this. The supplement has been revised and missing references were added.
Manuscript passage: S1 Appendix

Comment: *Supplement S1: where does the square root in (A.8) come from?*
Answer: There has been a mistake in the computation as the sum of the square roots needs to be squared. A justification of this step was added.
Manuscript passage: equation (A.8) and subsequent reasoning in S1 Appendix

Comment: *S1: the first inequality in the computation of $Var(J_A(s)/Y_A(s))$ should be justified.*
Answer: We thank the reviewer for this feedback. A justification of this inequality was added.
Manuscript passage: reasoning following the respective inequality

4 Comments of Reviewer #3

Comment: *No reference is made to the standard error of the estimates of type I error probability and power in Tables 1 and 2.*
Answer: We have specified the breadth of 95% confidence intervals for two different rates for the given number of simulation runs. The rates obtained in the

simulation have a similar magnitude to those two rates.
Manuscript passage: l. 167-169
