# Supplementary material for: Reference curve sampling variability in one–sample log–rank tests
Source: PLoS One. 2022 Jul 21;17(7):e0271094. doi: 10.1371/journal.pone.0271094 (PMC9302761; doi:10.1371/journal.pone.0271094)
Supplement: Attachment — Submitted filename: comments_to_editor.pdf [file pone.0271094.s005.pdf]

Comments to the editors on the article

"Reference curve sampling variability in one-sample log-rank tests" (PONE-D-22-01927)

Dear associate editor prof. dr. Ralf Bender,

I found that the authors addressed most of the previous comments. I appreciate their careful revision. Still, there is a series of minor points that should be fixed before I could recommend to accept the paper. For now, I recommend "minor revision". Please see my detailed comments to the authors for the specifics.

With best wishes,
Dennis Dobler
