# Supplementary material for: Reference curve sampling variability in one–sample log–rank tests
Source: PLoS One. 2022 Jul 21;17(7):e0271094. doi: 10.1371/journal.pone.0271094 (PMC9302761; doi:10.1371/journal.pone.0271094)
Supplement: Attachment — Submitted filename: review.pdf [file pone.0271094.s006.pdf]

Review of "Reference curve sampling variability in one-sample log-rank tests" (PONE-D-22-01927)

General: I appreciate the careful revision and replies to all reviewer comments. I am particularly fond of the discussion about potential confounding; this is indeed a very important point. I still have some minor comments that should be addressed before the paper is in an acceptable shape.

1. p. 4, ll. 88-92: even though it was already addressed in the peer-to-peer reply, I still think the definition of Z_{OSLR} should be adjusted (or one should add a word of caution into the manuscript): since the time horizon $[0, s_{max}]$ as given in the null hypothesis is fixed, it could well be that the cumulative hazards differ only after s_{max}. As a consequence, the null hypothesis could be true although the cumulative hazards are actually different (after s_{max}). Hence, the type-I error probability of the test cannot be under control.

2. p. 4, l. 113: it is not immediate why n_A cancels out in the definition of Z.

3. The figure captions are included in the paper but the figures are placed at the end of the document. (If this is required by the journal, my comment can be ignored.)

4. Appendix A, p. 3, second to last paragraph: it should be pointed out that the explanation why the conditional probability in the integral can be bounded by something that is independent of x_B come only below. Otherwise, it is difficult to comprehend the logic of the proof.

   Also, the final convergence in the very same paragraph seems to be missing: I think the probability goes to 0.

5. Appendix A, p. 4, last paragraph: the reference [1] does not seem to have a Lemma 4.2. Please double-check the reference.

6. Same paragraph: the variance bound does not seem immediate to me. For instance, where does the additional rate $1/n_A$ come from? (That is, the power -3 instead of -2.)

7. Appendix A, p. 7, at the top: even though the approximations are motivated by first-order Taylor expansions, they are biased: for example, from Jensen's inequality one gets $\sqrt{E(\sqrt{R})} \leq \sqrt{E(R)}$. This should be pointed out. Alternatively, one could include higher order terms of the Taylor expansions to get more reliable approximations.
