# Supplementary material for: Reference curve sampling variability in one–sample log–rank tests
Source: PLoS One. 2022 Jul 21;17(7):e0271094. doi: 10.1371/journal.pone.0271094 (PMC9302761; doi:10.1371/journal.pone.0271094)
Supplement: Attachment — Submitted filename: report.pdf [file pone.0271094.s007.pdf]

Reference Curve Sampling Variability in One-Sample Log-Rank Tests

by Feld, Danzer, Faldum and Schmidt

The authors explain how a one-sample log-rank test may be used to test whether the survival distribution for a new sample is the same as a reference survival distribution based on historical data. They point out that the standard method of testing does not adjust for error in estimating the reference distribution and, thus, the probability of finding a difference when both the new sample and the historical data follow the same distribution is liable to be inflated above the nominal type I error rate. The main contribution of the paper is to quantify the possible type I error rate inflation.

The description of methods is rather technical. Since the hope is that applied statisticians will take notice of these results, a simpler description would be helpful. Can references to σ-algebras and filtrations be confined to the appendix?

I believe the main contribution of the paper is the set of results presented in Table 1. Thus, a clear discussion of the patterns in this table and their generalisability to other cases is paramount.

The discussion makes a curious jump on line 189. After a reference to Figure 1, which compares allocation ratios, there is a comment about the effects of length of the observation period. I think this must relate to Figure 3, the content of which is only described later.

Only a subset of the cases considered in Table 1 strike me as practically relevant. The premise in comparing with historical data is that there is a good volume of such data. Hence, allocation ratios $\pi \leq 0.5$ are most important, with added relevance as π decreases. A test should have reasonable power: my calculations indicate that over 60 events should be observed in group B in order to have power 0.8 to detect a hazard ratio of 0.7. For some of the cases with $n = 100$, the expected number of events in group B is 10 or fewer. For values of $\pi \leq 0.5$, only $n \in \{100, 500\}$ gives a reasonable power.

Since the log-rank test is rank-based, I would not expect that varying the Weibull parameter k should have a significant effect on the results, other than through

the total number of events observed. (One can transform time so that survival follows an exponential distribution on the transformed scale and it is only the interaction of the staggered entry times and cumulative hazard in the follow-up period that complicates matters a little.) Thus it is not surprising that results are quite similar for all values of k. Moreover, I believe there are grounds to claim wider applicability of these findings.

Another reason that type I error may differ from its target value is the inadequacy of the normal approximation when there are small numbers of events in group B. However, these are also the cases with low power, and so not of great interest anyway.

Based on the above considerations, I suggest that results for $k = 1$, $\pi \leq 0.5$, $n \in \{100, 500\}$ are “representative”. If investigators wish to know whether their own study will be similarly affected by the problem, they should consider the numbers of events in group B and in the historical data. As long as these numbers are reasonably high, they should expect the results for the relevant value of π to be applicable.

In trying to understand the origin of the values of α and R in Table 1, I considered the simpler setting where data are normally distributed and one wishes to compare the mean response on a new treatment to a mean value based on historical data. In this setting, a simple calculation leads to the formulae

$$R = \sqrt{1/(1+\pi)} \tag{1}$$

and

$$\alpha = 2[1 - \Phi\{R\,\Phi^{-1}(1-\alpha/2)\}].$$

The agreement with results in Table 1 for cases with $n \in \{100, 500\}$ is remarkable. The paper eventually presents the same relationship between R and α but does not give the simple expression (1) for R. A possible explanation for why this expression should be so accurate is that the log-rank test is an efficient test of the equality of two distributions against a proportional hazards alternative and, implicitly, provides an efficient estimate of the hazard ratio. Thus, large sample theory on semiparametric efficient estimators implies approximate normality and variance inversely proportional to sample size, just as in my simple example with normally distributed responses: see Scharfstein et al. (1997).

One further point for consideration is that the two forms of type I error in a two-sided test have very different implications. Rejecting the null hypothesis and declaring the new treatment to be superior is of prime importance. Thus, it is important that a one-sided test has the appropriate type I error rate and, if results are to be presented for two-sided tests, error rates in both tails should be presented. There are examples of tests for survival data where the overall two-sided error rate hits its a target level α but error rates in the two tails are not close to $\alpha/2$: see, for example, Barber & Jennison (1999). Although there is some symmetry in the one-sample log-rank test, there is not complete symmetry, so there are reasons that this issue should be addressed.

Reference

Barber, S., and Jennison, C. (1999). Symmetric tests and confidence intervals for survival probabilities and quantiles of censored survival data. *Biometrics*, **55**, 430–436.

Scharfstein, D. O., Tsiatis, A. A., and Robins, J. M. (1997). Semiparametric efficiency and its implication on the design and analysis of group-sequential studies. *Journal of the American Statistical Association*, **92**, 1342–1350.
