# Supplementary material for: Reference curve sampling variability in one–sample log–rank tests
Source: PLoS One. 2022 Jul 21;17(7):e0271094. doi: 10.1371/journal.pone.0271094 (PMC9302761; doi:10.1371/journal.pone.0271094)
Supplement: Attachment — Submitted filename: answers_to_comments.pdf [file pone.0271094.s008.pdf]

Answer to reviewer comments
concerning the manuscript 'Reference Curve Sampling Variability in One–Sample Log–Rank Tests' (PONE-D-22-01927)

May 25, 2022

We revised the manuscript, particularly following the very detailed comments of the three reviewers. We aimed to fully implement and consider their very helpful suggestions, as will be detailed below in our answers to the reviewer comments. All changes made are highlighted in red color in the "track changes" version of the manuscript.

1 Comments of Reviewer #1

1. The Time-window for the analysis

   (a) Comment: The authors decided not the present the actual conditions on $s_{\max}$, the upper limit of the analysis window, but prefer a verbal description. "In particular, we require the time window of observation in the interventional group to be smaller than that of the control group". Maybe the authors can convince me from the opposite but this is not the same as the condition in Theorem 1 and 2 in the appendix. There it is said that (a) $S_{X_A}(s_0) = S_{T_A}(s_0)S_{C_A}(s_0) > 0$ is required. Here, the censoring also plays an important role. Does the authors suppose that the censoring in both groups are the same? Otherwise, I do not see how the upper verbal conditions implies the formal condition (a).
   Answer: We agree with the reviewer that our verbal description is not straightforwardly linked to the formal condition required in Theorem 1. We now clarified the connection in the manuscript.
   Manuscript passage: p.4, l.120 - p.5, l.124

   (b) Comment: In their response to my former comment, the authors state "Nevertheless, we have kept the formal argument "∞" in formulas (5), (7) and (8) to show that no data from any group is discarded in the final analysis if the condition addressed is complied with."
   From a theoretical point of view, the time window must be specified in advance before the data is collected. Since $s_{\max}$ need to be chosen

such that $P[X > s_{\max}] > 0$, it is expected that there are also observations larger than $s_{\max}$. These observations are not completely excluded because they are needed in the calculation for the Nelson-Aalen estimator. I understand that the restriction to $s_{\max}$ is not so nice, especially in the formulas, but it is what the theory gives you. Thus, I would prefer that it is mentioned appropriately in the paper. The restriction to $s_{\max}$ is also important for the so-called restricted mean survival time (RMST, see e.g. several papers of Royston). So it is not completely new and also accepted (at least from my point of view). There are also some discussion how to avoid the restrictions, see Tian et al (2020) for the RMST or Wang (1987) & Stute and Wang (1993) for uniform consistency of the Kaplan–Meier, but in both cases conditions on the censoring distribution are needed.

<u>Answer:</u> Thank you for your comment. Initially we wanted to keep the argument "∞" because the phenomenon of variance explosion may only be of theoretical nature in the limit (see answer to the next comment). In the case of $a_B + f_B < a_A + f_A$ the test statistic is constant after $s_{\max} = a_B + f_B$ and thus the arguments would be synonym. However, the reviewer is correct that our theorems only cover the argument $s_{\max}$ fulfilling the conditions of Theorem 1 and therefore we should use it in the definition of the test statistic instead.

<u>Manuscript passage:</u> p.4, eq. (6) and (7), p.4, l.120 - p.5, l.124

(c) <u>Comment:</u> How is $s_{\max}$ chosen in the data example and for the simulations? The conditions on $s_{\max}$ ensure that the variance does not "explode", right? Is there a guarentee that this does not happen in the simulations? When this is the case but no $s_{\max}$ is chosen, this would also be fine for me when the authors appropriately explain it in the respective section.

<u>Answer:</u> In the simulation study, the distribution of the censoring variable C is the same in both groups as the duration of the accrual period a and the duration of the follow-up period f are the same. In order to comply with the theoretical requirements, we chose $s_{\max} = a + f - 10^{-8}$ here. In our application example, we only considered combinations of a_B and f_B such that $a_B + f_B \leq 12$. As there are censored survival times with a value greater than 12, we can choose $s_{\max} = a_B + f_B$ there to include any observation from group B.

In general, an observation time in group B, which is greater than all observations of group A would have to be penalized with an additional variance term of magnitude $\mathcal{O}(n_A)$. This explodes if n_A approaches infinity. But in a finite sample case this is unproblematic. However, the theory does not cover this case. Nevertheless it is clear, that the underestimation of variance only becomes worse if such observations are made in group B. In this case, a restriction to

an interval $[0, s_{\max}]$ could be used to obtain a lower bound.
Manuscript passage: p.6, ll.184-185, p.11, ll.331-333

2. Comment: Minor Remark/Question: The log-rank test is known to be optimal for proportional hazard alternatives. But when early or late differences are expected including an appropriate weight in the statistic can lead to a siginifcant benefit in terms of power. I wonder whether such weights can also be implemented in the new proposal. When there is a quick solution, the authors may add a small discussion to the paper, otherwise please ignore the comment.
Answer: Thank you for your comment. Our results can well be extended to weighted testing procedures which are better suited to detect early or late differences. We included a corresponding outlook in the discussion section of the manuscript on how such extensions may be accomplished.
Manuscript passage: p.13, ll.412-417

2 Comments of Reviewer #2

1. Comment: p. 4, ll. 88-92: even though it was already addressed in the peer-to-peer reply, I still think the definition of Z_{OSLR} should be adjusted (or one should add a word of caution into the manuscript): since the time horizon $[0, s_{\max}]$ as given in the null hypothesis is fixed, it could well be that the cumulative hazards differ only after $s_{\max}$. As a consequence, the null hypothesis could be true although the cumulative hazards are not actually different (after $s_{\max}$). Hence, the type I error probability of the test cannot be under control.
Answer: Thank you for the comment. We adjusted the definition of the test-statistic Z_{OSLR} by changing the argument to $s_{\max}$.
Manuscript passage: p.5, eq. (7)

2. Comment: p. 4, l. 113: it is not immediate why n_A cancels out in the definition of Z.
Answer: To make it more clear, we added a short explanation why the constants n_B and n_A cancel out.
Manuscript passage: p.5, ll.125.127

3. Comment: The figure captions are included in the paper but the figures are placed at the end of the document. (If this is required by the journal, my comment can be ignored.)
Answer: If we understand the journal guidelines correctly, this is a requirement.

4. Comment: Appendix A, p. 3, second to last paragraph: it should be pointed out that the explanation why the conditional probability in the integral can be bounded by something that is independent of x_B come

only below. Otherwise, it is difficult to comprehend the logic of the proof. Also, the final convergence in the very same paragraph seems to be missing: I think the probability goes to 0.
Answer: We added a note in the proof that the required uniform bound is proven below.
Indeed, the mentioned probability goes to zero. We thank the reviewer for this correction.
Manuscript passage: S1 Appendix, p.4, first paragraph

5. Comment: Appendix A, p. 4, last paragraph: the reference [1] does not seem to have a Lemma 4.2. Please double-check the reference.
Answer: The reference is indeed not correct. We want to refer to Lemma 4.2 of [2].
Manuscript passage: S1 Appendix, p.5, first paragraph

6. Comment: Same paragraph: the variance bound does not seem immediate to me. For instance, where does the additional rate $1/n_A$ come from? (That is, the power -3 instead of -2.)
Answer: The additional rate $1/n_A$ is a result of Lemma 4.2 of [2] which was concerned in the previous comment.
Manuscript passage: S1 Appendix, p.5, first paragraph

7. Comment: Appendix A, p. 7, at the top: even though the approximations are motivated by first-order Taylor expansions, they are biased: for example, from Jensen's inequality one gets $E[\sqrt{R}] \leq \sqrt{E[R]}$. This should be pointed out. Alternatively, one could include higher order terms of the Taylor expansions to get more reliable approximations.
Answer: The reviewer is correct about the Taylor expansion. Our main goal was to provide a rule of thumb which is reasonable to compute for practitioners and therefore neglected higher order terms. Nevertheless we included his remark in the manuscript for the advanced reader.
Manuscript passage: S1 Appendix, p.8, first paragraph

3 Comments of Reviewer #3

1. Comment: The description of methods is rather technical. Since the hope is that applied statisticians will take notice of these results, a simpler description would be helpful. Can references to σ-algebras and filtrations be confined to the appendix?
Answer: As the reviewer suggested, we shifted the definition of the σ-algebras to the Appendix and gave a verbal definition instead.
Manuscript passage: p.3, ll.75-77 resp. S1 Appendix, p.1, first paragraph

2. Comment: The discussion makes a curious jump on line 189. After a reference to Figure 1, which compares allocation ratios, there is a comment about the effects of length of the observation period. I think this must relate to Figure 3, the content of which is only described later.

Answer: Thank you for the comment. We restructured our text in the corresponding section to make it more easy to follow. Actually, the passage mentioned in this comment also concerns Figure 1 as we displayed the type I error rate inflation for several choices of the follow-up period in the experimental cohort there. We hope that this gets more clear in the restructured version.
Manuscript passage: p.8, ll.250-257

3. Comment: Only a subset of the cases considered in Table 1 strike me as practically relevant. The premise in comparing with historical data is that there is a good volume of such data. Hence, allocation ratios $\pi \leq 0.5$ are most important, with added relevance as π decreases. A test should have reasonable power: my calculations indicate that over 60 events should be observed in group B in order to have power 0.8 to detect a hazard ratio of 0.7. For some of the cases with $n = 100$, the expected number of events in group B is 10 or fewer. For values of $\pi \leq 0.5$, only $n \in \{100, 500\}$ gives a reasonable power.
   Answer: We agree with the reviewer that the relevance is higher for small values of π. However we wanted to show also some extreme cases for validation of our theoretical results and highlighting effects in extreme cases. However, the relevance of sample sizes is highly dependent on the clinical background of the practitioner. Our background for example are studies in the field of pediatric oncology, where rather small sample sizes ($n_B \leq 50$) are also common.
   Manuscript passage: Section "Simulation study: effective type I error rate of the one-sample log-rank test"

4. Comment: Since the log-rank test is rank-based, I would not expect that varying the Weibull parameter κ should have a significant effect on the results, other than through the total number of events observed. (One can transform time so that survival follows an exponential distribution on the transformed scale and it is only the interaction of the staggered entry times and cumulative hazard in the follow-up period that complicates matters a little.) Thus it is not surprising that results are quite similar for all values of κ. Moreover, I believe there are grounds to claim wider applicability of these findings.
   Answer: Thank you for your comment. The reviewer is correct with his remark regarding the lesser importance of the shape parameter κ. We therefore shifted results for varying shape parameters $\kappa \neq 1$ to the Appendix and reduced the number of different values for κ used in the simulations. We furthermore included the analytical argument regarding kappa's subordinate role in the manuscript.
   Manuscript passage: Section "Simulation study: effective type I error rate of the one-sample log-rank test", in particular p.8, ll.228-234 and S1 Appendix, pp.9-11

5. Comment: Based on the above considerations, I suggest that results for

$\kappa = 1, \pi \leq 0.5, n \in \{100, 500\}$ are "representative". If investigators wish to know whether their own study will be similarly affected by the problem, they should consider the numbers of events in group B and in the historical data. As long as these numbers are reasonably high, they should expect the results for the relevant value of π to be applicable.
Answer: Thank you for your comment. We restructured the results table such that readers can find representative scenarios for their purposes more easily. Regarding the relevance of sample sizes, see our answer to comment 3.
Manuscript passage: as above

6. Comment: In trying to understand the origin of the values of α and R in Table 1, I considered the simpler setting where data are normally distributed and one wishes to compare the mean response on a new treatment to a mean value based on historical data. In this setting, a simple calculation leads to the formulae
$$R = \sqrt{1/(1+\pi)} \quad (1)$$
and
$$\alpha = 2(1 - \Phi(R\Phi^{-1}(1 - \alpha/2)))$$
The agreement with results in Table 1 for cases with $n \in \{100, 500\}$ is remarkable. The paper eventually presents the same relationship between R and α but does not give the simple expression (1) for R. A possible explanation for why this expression should be so accurate is that the log-rank test is an efficient test of the equality of two distributions against a proportional hazards alternative and, implicitly, provides an efficient estimate of the hazard ratio. Thus, large sample theory on semiparametric efficient estimators implies approximate normality and variance inversely proportional to sample size, just as in my simple example with normally distributed responses: see Scharfstein et al. (1997).
Answer: We thank the reviewer for his thorough investigation of this issue. We are very sorry that we did not investigate this relationship earlier because indeed formula (1) also applies here in a special case. While the general formula (see Appendix B) is valid in any situation, this relation simplifies to (1) under the null hypothesis in the case of equal censoring distribution for both groups. This is now also proven in Appendix B and shown in the main manuscript. As the censoring distributions in the two groups are equal in the simulation study, the estimated ratios $\widehat{R}$ converge against the respective values for increasing sample sizes.
Manuscript passage: p.5, ll. 138-145 and S1 Appendix, p.7

7. Comment: One further point for consideration is that the two forms of type I error in a two-sided test have very different implications. Rejecting the null hypothesis and declaring the new treatment to be superior is of prime importance. Thus, it is important that a one-sided test has the appropriate type I error rate and, if results are to be presented for two-sided

tests, error rates in both tails should be presented. There are examples of tests for survival data where the overall two-sided error rate hits its a target level α but error rates in the two tails are not close to $\alpha/2$ see, for example, Barber & Jennison (1999). Although there is some symmetry in the one-sample log-rank test, there is not complete symmetry, so there are reasons that this issue should be addressed.
<u>Answer:</u> Thank you for your comment. Indeed the one-sample log-rank test is quite unbalanced. We took this into account in our new simulations and present one-sided type I error rates in the results. Importantly, the choice of the variance estimator plays a role when it comes to the direction of this unbalancedness. The variance of the unstandardised one-sample log-rank test can basically be estimated with the counting process or its compensator. We present both possible choices in the manuscript and show the implications on the type I error rate inflation for small sample sizes of both of them in the simulation. Consequently, it is
<u>Manuscript passage:</u> p.4, ll.96-98 (introcution of both variance estimators), p.7, Table 2 and S1 Appendix, p.9, Table 2 and p.11, Table 5 (simulation results for one-sided tests)
